# A Phase 1, randomized, double-blind, placebo-controlled, single- and multiple-dose escalation study to evaluate the safety and pharmacokinetics/pharmacodynamics of PF-06835375, a C-X-C chemokine receptor type 5 directed antibody, in patients with systemic lupus erythematosus or rheumatoid arthritis

**DOI:** 10.1186/s13075-024-03337-2

**Published:** 2024-06-06

**Authors:** Stanley Cohen, Jean S. Beebe, Vishala Chindalore, Shunjie Guan, Mina Hassan-Zahraee, Madhurima Saxena, Li Xi, Craig Hyde, Sarita Koride, Robert Levin, Shannon Lubaczewski, Mikhail Salganik, Abigail Sloan, Erin Stevens, Elena Peeva, Michael S. Vincent, David A. Martin, Myron Chu

**Affiliations:** 1 Internal Medicine, University of Texas Southwestern Medical School Metroplex Clinical Research Center, 8144 Walnut Hill Lane, Suite 800, Dallas, TX 75231 USA; 2 Pfizer Inc, Cambridge, MA USA; 3 Pinnacle Research Group, LLC, Anniston, AL USA; 4 Clinical Research of West Florida, Clearwater, FL USA; 5 Pfizer Inc, Collegeville, PA USA; 6 Pfizer Inc, Groton, CT USA

**Keywords:** CXCR5, PF-06835375, Safety, Efficacy, Systemic lupus erythematosus, Rheumatoid arthritis, B-cell depletion, Follicular helper T cells depletion, Pharmacokinetics, Pharmacodynamics

## Abstract

**Background:**

The objective of this study was to evaluate the safety, tolerability, pharmacokinetics, and pharmacodynamics of PF‑06835375, a potent selective afucosyl immunoglobulin G1 antibody targeting C-X-C chemokine receptor type 5 (CXCR5) that potentially depletes B cells, follicular T helper (Tfh) cells, and circulating Tfh-like (cTfh) cells, in patients with systemic lupus erythematosus (SLE) and rheumatoid arthritis (RA).

**Methods:**

This first-in-human, multicenter, double-blind, sponsor-open, placebo-controlled Phase 1 study recruited patients aged 18–70 years with SLE or RA. In Part A, patients received single doses of intravenous PF-06835375 (dose range: 0.03–6 mg) or placebo in six sequential single ascending dose (SAD) cohorts. In Part B, patients received repeat doses of subcutaneous PF-06835375 (dose range: 0.3–10 mg) or placebo on Days 1 and 29 in five multiple ascending dose (MAD) cohorts. Tetanus/Diphtheria (Td) and Meningococcal B (MenB/Trumenba™) vaccines were administered at Day 4 (Td and MenB) and Week 8 (MenB only) to assess PF-06835375 functional effects. Endpoints included treatment-emergent adverse events (TEAEs), pharmacokinetic parameters, pharmacodynamic effects on B and cTfh cells, and biomarker counts, vaccine response, and exploratory differential gene expression analysis. Safety, pharmacokinetic, and pharmacodynamic endpoints are summarized descriptively. The change from baseline of B and Tfh cell-specific genes over time was calculated using a prespecified mixed-effects model, with a false discovery rate < 0.05 considered statistically significant.

**Results:**

In total, 73 patients were treated (SAD cohorts: SLE, *n* = 17; RA, *n* = 14; MAD cohorts: SLE, *n* = 22; RA, *n* = 20). Mean age was 53.3 years. Sixty-two (84.9%) patients experienced TEAEs (placebo *n* = 17; PF-06835375 *n* = 45); most were mild or moderate. Three (9.7%) patients experienced serious adverse events. Mean t_1/2_ ranged from 3.4–121.4 h (SAD cohorts) and 162.0–234.0 h (MAD cohorts, Day 29). B and cTfh cell counts generally showed dose-dependent reductions across cohorts (range of mean maximum depletion: 67.3–99.3%/62.4–98.7% [SAD] and 91.1–99.6%/89.5–98.1% [MAD], respectively). B cell-related genes and pathways were significantly downregulated in patients treated with PF-06835375.

**Conclusions:**

These data support further development of PF-06835375 to assess the clinical potential for B and Tfh cell depletion as a treatment for autoimmune diseases.

**Trial registration:**

ClinicalTrials.gov identifier: NCT03334851.

**Supplementary Information:**

The online version contains supplementary material available at 10.1186/s13075-024-03337-2.

## Introduction

Systemic lupus erythematosus (SLE) and rheumatoid arthritis (RA) are systemic autoimmune diseases with substantial morbidity and mortality [1–4]. The global prevalence of SLE and RA has been estimated at 43.7 per 100,000 and 246.6 per 100,000 individuals, respectively [2, 5]. The prevalence of both SLE and RA varies by global region, although a trend of increasing prevalence has been reported over the last few decades [2, 5].

Treatment options for patients with SLE and RA have improved over the last two decades, with a better understanding of the pathobiology of the diseases, coupled with the development of targeted therapies, such as cytokine inhibitors and Janus kinase inhibitors for RA, and B cell and Type 1 interferon-directed therapy for SLE [6–9]. Even with improved treatments, 37% of patients with RA continue to experience active disease despite therapy [10], and a large proportion of patients with SLE still require disease-modifying antirheumatic drugs [11], despite their toxicity [11], and corticosteroids [11]. Given the risks associated with the long-term use of many existing treatments for SLE and RA, and that they are frequently insufficient to adequately control active disease [11, 12], there remains a need for safer and more effective therapies that can induce long-lasting remission.

B cell and T cell dysregulation is involved in the pathology of SLE and RA [13–15]. In SLE, T cells amplify inflammation by secreting pro-inflammatory cytokines, thus mediating autoantibody production by B cells, which in turn maintain disease through the accumulation of autoreactive memory T cells [16]. Meanwhile, in RA, B cells secrete proteins implicated in disease pathogenesis, such as rheumatoid factors, anti-citrullinated protein antibodies, and pro-inflammatory cytokines [17]. A major role of T cells in RA is to activate macrophages and fibroblasts, leading to the production of various cytokines and chemokines which exacerbate joint inflammation [17]. Follicular T helper (Tfh) cells are also implicated in the pathogenesis of autoimmune diseases, due to their role in the germinal center reaction, affinity maturation, and autoantibody generation [18, 19], and are elevated in both SLE and RA [20, 21]. The dysregulation of these immune cell types, and their involvement in the pathogenesis of SLE and RA, suggests therapeutic potential for targeting B and Tfh cells together in autoimmune diseases in order to inhibit the production of autoantibodies targeting self-antigens.

PF-06835375 is a humanized, afucosyl immunoglobulin G1 antibody selective against C-X-C chemokine receptor type 5 (CXCR5) expressed on B cells, Tfh cells, and circulating Tfh-like (cTfh) cells. PF-06835375 is in development for the treatment of autoimmune diseases through depletion of CXCR5-positive B and Tfh cells and antagonism of C-X-C motif chemokine ligand 13-dependent signaling, thereby representing a new strategy for treating SLE and RA.

This first-in-human study (NCT03334851) evaluated the safety, tolerability, pharmacokinetics (PK), and pharmacodynamics (PD) of PF‑06835375 in patients with seropositive SLE and RA, and assessed the functional effect of PF-06835375 on vaccine responses in this pool of autoimmune patients.

## Methods

### Patients

Patients were eligible for inclusion if they were aged 18–70 years, with a body mass index of 17.5–40 kg/m^2^, and a total body weight > 45 kg. In this Phase 1 study, patients were required to have either seropositive SLE or RA but without minimum disease activity requirement for inclusion.

SLE diagnosis was confirmed using the Systemic Lupus International Collaborating Clinics classification criteria and a positive anti-nuclear antibodies titer ≥ 1:80, and/or anti-dsDNA, and/or anti-Smith antibodies at screening. Patients with a clinical Systemic Lupus Erythematosus Disease Activity Index 2000 (SLEDAI-2K) score > 8 were excluded from the three lowest single dose cohorts in Part A of the study but were permitted to enroll in all other cohorts in Parts A and B, following consultation with the medical monitor.

RA diagnosis was confirmed using the 2010 American College for Rheumatology/European Alliance of Associations for Rheumatology criteria and positive rheumatoid factor and/or anti-citrullinated peptide antibody. Patients with a Disease Activity Score 28 score > 5.1 were excluded from the three lowest single dose cohorts in Part A of the study but were permitted to enroll in all other cohorts in Parts A and B, following consultation with the medical monitor.

The use of certain non-prescription concomitant treatments was permissible during the study, although patients were asked to abstain from initiating new treatments. All concomitant treatments taken during the study were recorded with indication, daily dose, and start and stop dates of administration. Concomitant treatments were recorded at each clinic visit.

### Study design

The study was a multicenter, randomized, double-blind, sponsor-open, placebo-controlled Phase 1 single and multiple-dose escalation study consisting of two parts. The study design is illustrated in Fig. S1.

In Part A, patients with SLE or RA were randomized 3:3:3:3:6:5 to intravenous (IV) PF-06835375 (0.03, 0.1, 0.3, 1, 3, or 6 mg) in six sequential single ascending dose (SAD) cohorts. Nine patients were randomized to placebo across the six SAD cohorts. Patients randomized in Part A were not eligible to enroll in Part B. In Part B, patients with SLE or RA were randomized 6:6:7:6:6 to subcutaneous (SC) PF-06835375 (0.3, 1, 3, 6, or 10 mg) administered on Days 1 and 29 in five multiple ascending dose (MAD) cohorts. Eleven patients were randomized to placebo across the five MAD cohorts. Additional pre-dose medications, including corticosteroids, were administered based on emerging safety and tolerability data, and post-dose treatments were allowed at the investigator’s discretion.

In Part A, 100 mg IV methylprednisolone was administered pre-dose to all patients in the cohort receiving PF-06835375 6 mg IV to mitigate the potential for infusion-related reactions. In Part B, oral prednisone 40 mg pre-dose and 20 mg post-dose were administered to the remaining patients to be enrolled into the cohort receiving PF-06835375 3 mg SC, as the first patients who received this dose experienced infusion-related reactions. Oral prednisone 40 mg pre-dose and 20 mg post-dose were also administered to all patients in the cohorts receiving PF-06835375 6 mg and 10 mg SC.

The selected starting doses (SAD and MAD) were planned to provide predicted exposure margins of multiple thousands (> 10,000) compared to the exposure-stopping limit at no observed adverse effect level (NOAEL) while having minimal B-cell depletion. Maximum doses were planned such that at least a tenfold exposure margin compared to NOAEL exists with a maximal predicted B-cell depletion. MAD doses are higher compared to SAD doses, as the route of administration is different. SC SAD dosing has reduced absorption compared to IV MAD dosing, which reduces the amount of drug that reaches the systemic circulation.

To mitigate unanticipated safety risks, the study utilized sentinel dosing for cohorts of more than four patients. This involved dosing of additional patients following communication between the sponsor's medical monitor and investigators from sites at which sentinel patients were first enrolled.

In order to provide an understanding of PD properties related to the degree of B and cTfh cell depletion, the functional effects of PF-06835375 were assessed with two vaccine challenges. On Day 4, the tetanus/diphtheria (Td) vaccine was administered. On Day 4 and Week 8, the Meningococcal B (MenB/Trumenba™) vaccine was administered. The Td vaccine was used as a recall challenge as it induces clear secondary antibody and immune cell responses. The MenB vaccine was used as a neoantigen when administered at Day 4 and as a recall challenge when administered at Week 8.

All patients were followed for a minimum of 16 weeks after the last dose of PF-06835375 was administered; study completion criteria were based on B cell counts meeting ≥ 1 of the following criteria: (1) ≥ 50% of baseline value and stable or increasing between Weeks 12–16; (2) ≥ lower limits of normal (80 cells/µL) and stable or increasing between Weeks 12–16. Patients not meeting either criterion at Week 16, continued in the study until B cell counts met ≥ 1 of the criteria, or until B cell counts reached a new stable level and the patient was clinically stable for ≥ 3 consecutive visits, each at least two weeks apart, based on assessment by both the sponsor and the investigator.

The first visit of the first patient took place in November 2017 and the last visit of the last patient took place in February 2022, which encompassed the period during the COVID-19 pandemic.

This study was approved by the Schulman Associates Institutional Review Board, Inc. (reference number IORG000063) and was conducted in compliance with the ethical principles originating in or derived from the Declaration of Helsinki and all International Council for Harmonisation Good Clinical Practice guidelines and International Ethical Guidelines for Biomedical Research Involving Human Subjects (Council for International Organizations of Medical Sciences 2022. Written informed consent was required from each patient before any study-specific activity.

### Endpoints

#### Primary endpoints

The primary objectives of the study were to examine the safety and tolerability of PF-06835375. Primary endpoints were the incidence of dose-limiting or treatment-related treatment-emergent adverse events (TEAEs); incidence, severity, and causal relationship of TEAEs and withdrawals due to TEAEs; incidence of chemistry, hematology, and urinalysis laboratory findings through the end of the study; abnormal and clinically relevant changes in vital signs and electrocardiogram (ECG) parameters; and incidence of infections.

#### Secondary pharmacokinetic and pharmacodynamic endpoints

Serum PF-06835375 concentrations after single (Day 1 IV SAD cohorts) and multiple (Day 1 and 29 SC MAD cohorts) PF‑06835375 doses were determined. PK parameters were generated by noncompartmental methods.

For SAD cohorts (Part A), the following PK parameters were analyzed: area under the concentration–time profile from time 0 extrapolated to infinite time (AUC_inf_); area under the concentration–time profile from time 0 to the time of the last quantifiable concentration (AUC_last_), maximum serum concentration (C_max_); time at which C_max_ occurred (T_max_), terminal half-life (t_½_), and clearance (CL).

For MAD cohorts (Part B), the following PK parameters were analyzed: area under the concentration–time profile from Day 1 to Day 29 (AUC_tau_), AUC_last_, C_max_, T_max_, t_½_, and apparent clearance (CL/F).

The absolute count of circulating CXCR5-positive B cells and cTfh cells over time was assessed for the SAD and MAD cohorts following administration of PF-06835375. T cell and B cell absolute counts were determined with flow cytometry using BD Multitest™ 6-color TBNK with BD Trucount™ tubes according to the manufacturer’s instructions (BD Biosciences, San Jose, CA). Data were acquired and analyzed with a FACSCantoII cytometer using BD FACSCanto Clinical Software. In addition, a flow cytometry panel that monitored T helper cell subsets, including CD3^+^CD4^+^CD45RO^+^CXCR5^+^ cTfh, was also performed. Whole blood was stained with an antibody cocktail containing anti-human monoclonal antibodies against CD45-AF700 and CCR6-BV421 (Biolegend, San Diego, CA), CD3-APC-H7, CD4 PerCP-Cy5.5, CD45RO-BV510, PD1-PE, ICOS-AF647, CD183-PE-Cy7 (BD Biosciences), and a non-drug competitive CXCR5-AF488 antibody (Creative Diagnostics, Shirley, NY), for 20 min at room temperature. Red blood cells were lyzed using FACSLyse (BD Biosciences), washed with staining buffer, and data were immediately acquired on a BD FACSCanto II flow cytometer. The absolute count of CD4^+^ cTfh cells was calculated by multiplying the frequency of Tfh cells of total CD4 T cells by the absolute count of CD4^+^ T cells derived from the BD Multitest™ Trucount™ assay. Depletion was defined as cell counts below 10 cells/μL. The incidence of the development of anti-drug antibodies (ADAs) and neutralizing antibodies (NAbs) were also examined.

#### Tertiary and exploratory pharmacodynamic and biomarker endpoints

The geometric means of B cell activating factor (BAFF) at baseline, as well as the geometric means of antibody response to the Td and MenB vaccines were assessed over time.

Whole blood collected in PAXgene tubes (BD Biosciences) was used to generate RNA sequencing (RNA-seq) datasets from Day 1 (pre-dose), 29 (pre-dose), 57, and 113. Samples collected from patients in all treatment groups in the SC MAD cohort and from patients in the 6 mg IV SAD cohort were used for data generation and analysis. Briefly, globin-depleted RNA was used for library preparation using the TruSeq Stranded mRNA Library Prep Kit (Illumina) and sequenced via next-generation sequencing on the Illumina NovaSeq 6000 platform. All samples were sequenced using paired-end 100 bp reads and mapped to the human genome assembly GRCh38. Samples were analyzed for the following genes: CD19, CXCR5, interleukin (IL)-6, and tumor necrosis factor (TNF) receptor superfamily members (TNFRSF)13B (TNFRSF13B), 13C (TNFRSF13C), and 17 (TNFRSF17).

### Statistical analyses

Safety data and PK parameters were presented using descriptive summary statistics.

R programming language (www.r-projects.com) [22] was used to perform all transcriptomic analyses. The transcriptomic data were modeled longitudinally using a mixed-effects model with random subject effect, fixed treatment, and visit effects, using log_2_ counts per million (CPM) values following trimmed mean of M-values normalization [23] using the R package limma [24]. Due to minimal variance observed in a principal component analysis, samples from the SAD and MAD placebo cohorts were pooled into a single placebo group for statistical analysis. The Benjamini–Hochberg procedure [25] was used to adjust p-values for multiple hypotheses by controlling the false discovery rate (FDR).

Gene set analysis was performed using Fast Gene Set Enrichment Analysis (FGSEA) method [26] implemented within the Gene Set Enrichment Analysis package and the Molecular Signatures Database (mSigDB). A pre-ranked list of genes based on estimates of the summary statistics from a mixed-effects model was used as the input for pathway analysis with a significance of *p* < 0.05. Sample level log_2_ CPM was calculated for the selected significant gene sets. Modeling was performed using the same approach described for single genes [27]. The change from baseline of B and Tfh cell-specific genes over time was calculated using the mixed-effects model described previously. Differentially expressed genes for any post- versus pre-treatment comparison with FDR < 0.05 were considered statistically significant.

A total of four different analysis populations were employed in this study. The PK analysis population included all patients who received at least one dose of the study treatment and had evaluable PK data. The PD analysis population included all patients who received at least one dose of the study treatment and had at least one PD measurement. The safety analysis population included all patients who received at least one dose of the study treatment. The pooled-placebo analysis population included all patients enrolled in the placebo groups, regardless of SAD or MAD cohorts.

## Results

### Patients

**Fig. 1:**
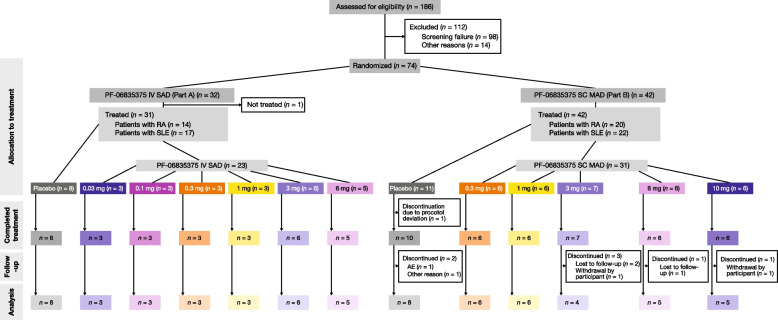
Patient disposition in single ascending dose and multiple ascending dose cohorts. *AE* adverse event, *IV* intravenous, *MAD* multiple ascending dose, *RA* rheumatoid arthritis, *SAD* single ascending dose, *SC* subcutaneous, *SLE* systemic lupus erythematous

Patient disposition is illustrated in Fig. 1. In total, 74 patients were randomized and 73 patients were treated (SAD cohorts: SLE, *n* = 17; RA, *n* = 14; MAD cohorts: SLE,* n* = 22; RA, *n* = 20). Of patients receiving placebo in the MAD cohort, one discontinued due to a protocol deviation, and two patients discontinued during the follow-up phase; of patients receiving PF-06835375 3 mg SC, three discontinued during follow-up; of patients receiving PF-06835375 6 mg and 10 mg, one in each group discontinued during follow-up. Pre- and post-dose corticosteroids were administered in the PF-06835375 3 and 6 mg IV cohorts and 3, 6, and 10 mg SC cohorts. Patient demographics and baseline characteristics are presented in Table 1. Mean (standard deviation) age was 53.3 (10.7) years. Most patients were female (n = 65, 89.0%) and White (n = 54, 74.0%). At baseline, the mean SLEDAI-2K and Disease Activity Score 28 C-reactive protein values were 0.58 and 0.46, in patients with SLE and RA, respectively.

**Table 1:** Patient demographics and baseline characteristics (safety analysis population)

	**All patients in the SAD cohorts** **(*N* = 31)**	**All patients in the MAD cohorts** **(*N* = 42)**
Age, years; mean (SD)	52.3 (10.4)	54.0 (11.0)
Female, *n* (%)	29 (93.5)	36 (85.7)
Race, *n* (%)		
White	19 (61.3)	35 (83.3)
Black or African American	11 (35.5)	4 (9.5)
Asian	0	1 (2.4)
American Indian or Alaska	0	1 (2.4)
Native	0	0
Native Hawaiian or Other Pacific Islander		
Other	0	0
Unknown multiracial	1 (3.2)	0
Not reported	0	1 (2.4)
Ethnicity, *n* (%)		
Not Hispanic or Latino	27 (87.1)	36 (85.7)
Weight, kg; mean (SD)	74.8 (16.1)	85.3 (14.7)
BMI, kg/m^2^; mean (SD)	28.4 (5.7)	31.6 (4.9)
Disease duration, years; mean (SD)^a^		
Rheumatoid arthritis	14.1 (10.1)	14.2 (11.2)
Systemic lupus erythematosus	9.3 (9.8)	10.6 (11.9)

The safety analysis population included all patients who received at least one dose of the study treatment

*BMI* body mass index, *MAD* multiple ascending dose, *SAD* single ascending dose, *SD* standard deviation

^a^Calculated based on the number of patients with the specified disease in the SAD or MAD cohort

### Primary safety endpoints

A total of 62 (84.9%) patients across the SAD and MAD cohorts experienced TEAEs (Table 2), most of which were mild or moderate in severity. A total of three (9.7%) patients (all in the SAD cohort) experienced serious adverse events (SAEs) unrelated to the study treatment, and one (9.1%) patient discontinued due to a TEAE of disease progression unrelated to the study treatment in the placebo SC cohort.

**Table 2:** Summary of incidence and severity of treatment-emergent adverse events (All causalities) (safety analysis population)

Number of patients, *n* (%)	SAD cohorts							MAD cohorts					
	**Placebo (*n* = 8)**	**PF-06835375 IV**						**Placebo (*n* = 11)**	**PF-06835375 SC**				
		**0.03 mg (*n* = 3)**	**0.1 mg (*n* = 3)**	**0.3 mg (*n* = 3)**	**1 mg (*n* = 3)**	**3 mg (*n* = 6)**	**6 mg (*n* = 5)**		**0.3 mg (*n* = 6)**	**1 mg (*n* = 6)**	**3 mg (*n* = 7)**	**6 mg (*n* = 6)**	**10 mg (*n* = 6)**
TEAEs	8 (100.0)	3 (100.0)	3 (100.0)	2 (66.7)	3 (100.0)	6 (100.0)	5 (100.0)	9 (81.8)	4 (66.7)	4 (66.7)	5 (71.4)	4 (66.7)	6 (100.0)
Mild	3 (37.5)	0 (0.0)	1 (33.3)	0 (0.0)	0 (0.0)	3 (50.0)	0 (0.0)	4 (36.4)	2 (33.3)	1 (16.7)	1 (14.3)	1 (16.7)	3 (50.0)
Moderate	4 (50.0)	2 (66.7)	2 (66.7)	2 (66.7)	3 (100.0)	3 (50.0)	4 (80.0)	5 (45.5)	2 (33.3)	3 (50.0)	4 (57.1)	3 (50.0)	3 (50.0)
Severe	1 (12.5)	1 (33.3)	0 (0.0)	0 (0.0)	0 (0.0)	0 (0.0)	1 (20.0)	0 (0.0)	0 (0.0)	0 (0.0)	0 (0.0)	0 (0.0)	0 (0.0)
Infections and infestations^a^	4 (50.0)	2 (66.7)	3 (100.0)	0 (0.0)	2 (66.7)	1 (16.7)	3 (60.0)	3 (27.3)	2 (33.3)	3 (50.0)	2 (28.6)	0 (0.0)	2 (33.3)
Laboratory abnormalities^b^	1 (12.5)	0 (0.0)	0 (0.0)	0 (0.0)	1 (33.3)	0 (0.0)	0 (0.0)	0 (0.0)	0 (0.0)	0 (0.0)	1 (14.3)	0 (0.0)	0 (0.0)
ECG abnormalities^c^	0 (0.0)	0 (0.0)	1 (33.3)	0 (0.0)	1 (33.3)	0 (0.0)	1 (20.0)	0 (0.0)	0 (0.0)	0 (0.0)	0 (0.0)	0 (0.0)	0 (0.0)
SAEs^d^	1 (12.5)	1 (33.3)	0 (0.0)	1 (33.3)	0 (0.0)	0 (0.0)	0 (0.0)	0 (0.0)	0 (0.0)	0 (0.0)	0 (0.0)	0 (0.0)	0 (0.0)
Discontinuations due to TEAEs^e^	0 (0.0)	0 (0.0)	0 (0.0)	0 (0.0)	0 (0.0)	0 (0.0)	0 (0.0)	1 (9.1)	0 (0.0)	0 (0.0)	0 (0.0)	0 (0.0)	0 (0.0)

The safety analysis population included all patients who received at least one dose of the study treatment

*ECG* electrocardiogram*, IV* intravenous, *MAD* multiple ascending dose, *SAD* single ascending dose, *SAE* serious adverse event, *SC* subcutaneous, *TEAE* treatment-emergent adverse event

^a^Infections and infestations reported as TEAEs in IV SAD: placebo (viral gastroenteritis [*n* = 1], sinusitis [*n* = 1], and upper respiratory tract infection [*n* = 2]) and PF-06835375 0.03 mg (upper respiratory tract infection [*n* = 1]and urinary tract infection [*n* = 1]), 0.1 mg (upper respiratory tract infection [*n* = 1] and urinary tract infection [*n* = 2]), 1 mg (bacterial vulvovaginitis [*n* = 1], viral gastroenteritis [*n* = 1], influenza [*n* = 1], tonsilitis [*n* = 1], and urinary tract infection [*n* = 1]), 3 mg (upper respiratory tract infection [*n* = 1]), and 6 mg (acute sinusitis [*n* = 1], otitis media [*n* = 1], upper respiratory tract infection [*n* = 1], viral upper respiratory tract infection [*n* = 2], and urinary tract infection [*n* = 1]); in SC MAD: placebo (gastrointestinal viral infection [*n* = 1], nasopharyngitis [*n* = 1], and otitis media [*n* = 1]) and PF-06835375 0.3 mg (herpes simplex [*n* = 1] and urinary tract infection [*n* = 1]), 1 mg (acute sinusitis [*n* = 1], urinary tract infection [*n* = 2], and viral upper respiratory tract infection [*n* = 1]), 3 mg (herpes simplex [*n* = 1], oral herpes [*n* = 1], and tooth abscess [*n* = 1]), and 10 mg (urinary tract infection [*n* = 1] and viral infection, *n* = 1])

^b^Laboratory abnormalities reported as TEAEs (placebo IV SAD: blood creatine phosphokinase increased, PF-06835375 1 mg IV SAD: hepatic enzyme increased, and PF-06835375 3 mg SC MAD: liver function test increased)

^c^ECG results reported as TEAEs (PF-06835375 0.1 mg IV SAD and PF-06835375 1 mg IV SAD: heart rate increased and PF-06835375 6 mg IV SAD: ECG T wave amplitude decreased)

^d^SAEs (placebo IV SAD: acute myocardial infarction, back pain, neck pain, and arteriosclerosis coronary artery, PF-06835375 0.03 mg IV SAD: chest pain, and PF-06835375 0.3 mg IV SAD: hypertension)

^e^Discontinuations due to TEAEs (placebo SC MAD: disease progression; unrelated to the study treatment)

In the SAD cohort, a total of 15 (48.4%) patients experienced treatment-related TEAEs: three (37.5%) patients receiving placebo IV, one (33.3%) patient receiving PF-06835375 0.1 mg IV, two (66.7%) patients receiving PF-06835375 1 mg IV, six (100.0%) patients receiving PF-06835375 3 mg IV, and three (60.0%) patients receiving PF-06835375 6 mg IV. In the MAD cohort, 16 (38.1%) patients experienced treatment-related TEAEs: three (27.3%) patients receiving placebo SC, three (50.0%) patients receiving PF-06835375 0.3 mg SC, one (16.7%) patient receiving PF-06835375 1 mg SC, five (71.4%) patients receiving PF-06835375 3 mg SC, two (33.3%) patients receiving PF-06835375 6 mg SC, and two (33.3%) patients receiving PF-06835375 10 mg SC.

The most common TEAEs in the SAD and MAD cohorts combined during the active collection period (from informed consent through at least 90 days after the last administration of PF-06835375) were headache (*n* = 18, 24.7%), pyrexia (*n* = 11, 15.1%), and urinary tract infection (*n* = 9, 12.3%). The most common infections after urinary tract infection were upper respiratory tract infection (*n* = 6, 8.2%) and viral upper respiratory tract infection (*n* = 3, 4.1%). All infections were mild or moderate in severity.

Two patients who both received PF-06835375 3 mg IV were reported to have mild infusion-related reaction, with onset at Day 1 and resolved at Day 14 and Day 15, and one patient who received PF-06835375 3 mg SC was reported to have mild allergic reaction, with onset at Day 1 and resolved at Day 2. All were deemed related to the study treatment. None of these events required treatment discontinuation.

Laboratory and ECG abnormalities (*n* = 3, each) considered clinically significant are shown in Table 2. All ECG abnormalities occurred in the SAD cohorts (PF-06835375 0.1, 1, and 6 mg). Of these ECG abnormalities, two patients experienced increased heart rate, whereas one patient experienced decreased ECG T wave amplitude. Each of these events were mild or moderate in severity and were resolved within the same day of dosing. No clinically significant changes in vital signs or deaths occurred.

Secondary pharmacokinetic and pharmacodynamic endpoints.

### Pharmacokinetic endpoints

**Fig. 2:**
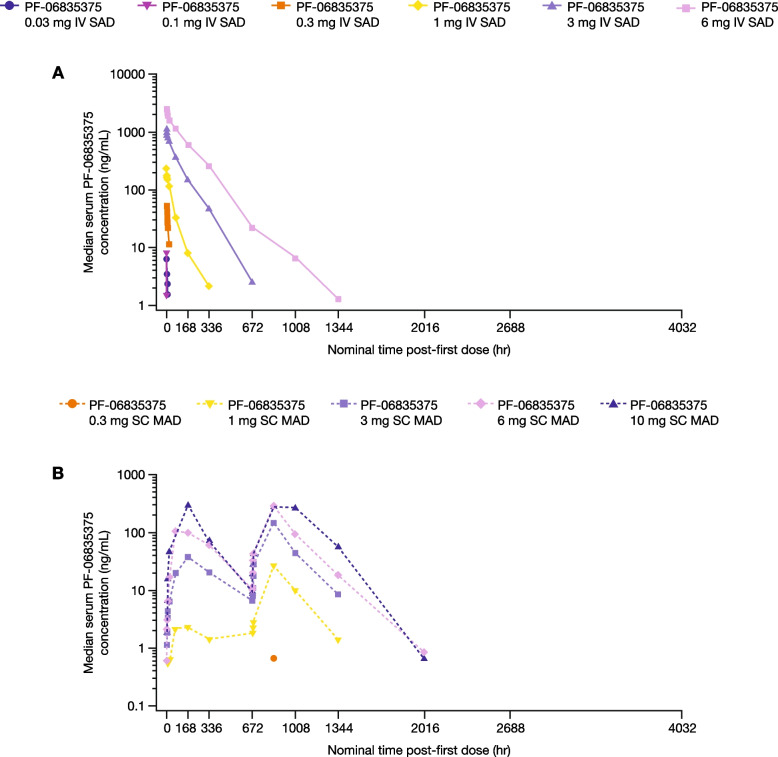
Serum PF-06835375 concentration–time profiles in the **A** single ascending dose and **B** multiple ascending dose cohorts (pharmacokinetic analysis population). The PK analysis population included all patients who received at least one dose of the study treatment and had evaluable PK data. The plots presented are semi-logarithmic. *IV* intravenous, *MAD* multiple ascending dose, *PK* pharmacokinetic, *SAD* single ascending dose, *SC* subcutaneous

PK parameters are shown in Tables 3 and 4; concentration–time profiles for SAD and MAD cohorts are shown in Fig. 2A and B, respectively. Among the SAD cohorts (Part A), median T_max_ ranged from 2–4 h and mean CL from 0.021–0.313 L/h. Mean t_1/2_ ranged from 3.40–121.4 h. Exposure (AUC and C_max_) generally appeared to increase dose-proportionally for doses ≤ 1 mg and more than dose-proportionally for doses > 1 mg. Among MAD cohorts, Day 1 median T_max_ ranged from 144–170 h; the increase in exposure (AUC_tau_ and C_max_) generally appeared to be greater than dose-proportional. Due to high affinity of the antibody to CXCR5 on the B-cell surface, significant target-mediated drug disposition at lower concentrations was observed. A reduction in clearance was also observed with higher doses and hence higher concentrations, which is indicative of target saturation and target-mediated drug disposition behavior. Day 29 median T_max_ ranged from 121–171 h and mean CL/F from 0.0785–0.117 L/h. Day 29 mean t_1/2_ ranged from 162.0–234.0 h. Exposure (AUC_tau_ and C_max_) generally appeared to be dose-proportional.

**Table 3:** Descriptive summary of serum PK parameters for PF-06835375 in IV SAD cohorts (PK analysis population)

PK parameters (unit)^a^	PF-06835375 IV SAD cohorts					
	**0.03 mg (*n* = 2)**	**0.1 mg (*n* = 1)**	**0.3 mg (*n* = 3)**	**1 mg (*n* = 3)**	**3 mg (*n* = 6)**	**6 mg (*n* = 5)**
AUC_inf_ (ng/h/mL), geometric mean (CV)	35.6, 95.7	-	1573 (2409)	7467 (84)	88,190 (35)	288,000 (30)
AUC_last_ (ng/h/mL), geometric mean (CV)	28.0, 67.9	39.1	1380 (2950)	7327 (85)	87,120 (36)	287,400 (30)
C_max_ (ng/mL), geometric mean (CV)	6.4, 7.4	10.8	103 (197)	208.7 (28)	994.7 (29)	2645 (13)
T_max_ (h), median (range)	2.0, 2.0	2.2	2.2 (2.2–2.2)	2.1 (2.1–4.2)	2.2 (2.2–4.1)	4.0 (2.0–8.1)
t_½_ (h), arithmetic mean (SD)	3.4, 12.8	-	35.1 (44.5)	52.5 (14.4)	91.4 (25.6)	121.4 (33.9)
CL (L/h), geometric mean (CV)	0.31, 0.84	-	0.19 (2420)	0.13 (84)	0.03 (35)	0.02 (29)

The PK analysis population included all patients who received at least one dose of the study treatment and had evaluable PK data

*AUC*_*inf*_ area under the concentration–time curve from zero to infinity, *AUC*_*last*_ area under the concentration–time curve from time zero to time of last quantifiable concentration, *CL* clearance, *C*_*max*_ maximum observed concentration, *CV* coefficient of variation, *IV* intravenous, *PK* pharmacokinetics, *SAD* single ascending dose, *SD* standard deviation, *t*_*½*_ terminal phase half-life, *T*_*max*_ time to first occurrence of C_max_

^a^Individual values are listed when there were less than three evaluable measurements

**Table 4:** Descriptive summary of serum PK parameters for PF-06835375 in SC MAD cohorts (PK analysis population)

PK parameters (unit)	PF-06835375 SC MAD cohorts							
	**Day 1**				**Day 29**			
	**1 mg (*n* = 5)**^**a**^	**3 mg (*n* = 7)**	**6 mg (*n* = 6)**	**10 mg (*n* = 6)**^**b**^	**1 mg (*n* = 5)**^**c**^	**3 mg (*n* = 7)**	**6 mg (*n* = 6)**	**10 mg (*n* = 6)**^**b**^
AUC_tau_ (ng/h/mL), geometric mean (CV)	1964 (130)	11,010 (84)	28,070 (251)^c^	70,630 (202)^c^	9309 (62)	25,610 (125)^e^	76,450 (142)	88,540 (154)
AUC_last_ (ng/h/mL), geometric mean (CV)	1671 (171)	11,080 (83)	28,760 (198)	68,280 (204)	9346 (61)	22,050 (146)	84,370 (160)	96,610 (141)
C_max_ (ng/mL), geometric mean (CV)	5.743 (148)	34.82 (79)	77.72 (150)	257.8 (204)	26.9 (68)	89.7 (93)	209.6 (115)	262.1 (224)
T_max_ (h), median (range)	170 (67.4–170)	168 (72.7–361)	169 (73.3–192)	144 (47.7–172)	169 (168–169)	169 (123–337)	171 (120–336)	121 (8–169)
t_½_ (h), arithmetic mean (SD)	-	-	-	-	234.0 (78.9)	162.0 (56.1)^a^	193.3 (62.8)	175.8 (52.8)^a^
CL/F (L/h)	-	-	-	-	0.11 (62)^c^	0.12 (125)^d^	0.08 (142)	0.11 (154)

The PK analysis population included all patients who received at least one dose of the study treatment and had evaluable PK data

*AUC*_*last*_ area under the concentration–time curve from time zero to time of last quantifiable concentration, *AUC*_*tau*_ area under the concentration–time curve overdosing interval, *CL/F* apparent clearance, *C*_*max*_ maximum observed concentration, *CV* coefficient of variation, *MAD* multiple ascending dose, *PK* pharmacokinetics, *SC* subcutaneous, *SD* standard deviation, *t*_*½*_ terminal phase half-life, *T*_*max*_ time to first occurrence of C_max_

^a^Only *n* = 4 contributed to the summary statistics

^b^Only *n* = 5 contributed to the summary statistics

^c^Only *n* = 3 contributed to the summary statistics

^d^Only *n* = 6 contributed to the summary statistics

### Pharmacodynamic endpoints

**Fig. 3:**
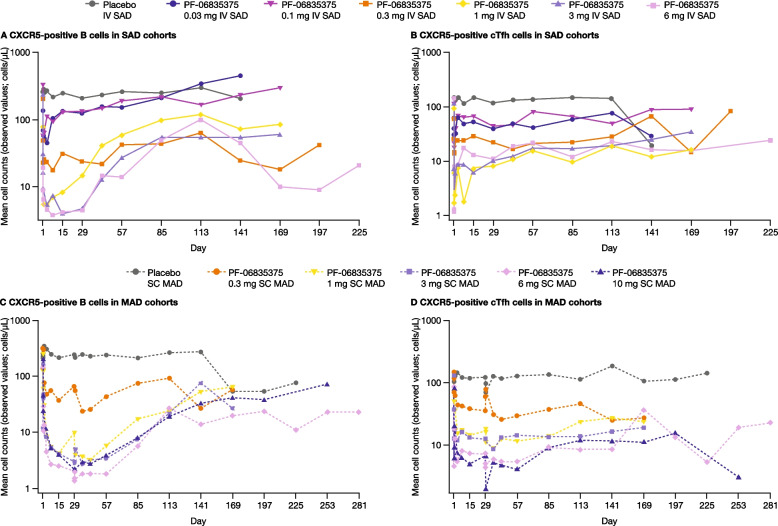
Mean **A**, **B** CXCR5-positive B and **C**, **D** cTfh cell counts in the single ascending dose and multiple ascending dose cohorts (PD analysis population). The PD analysis population included all patients who received at least one dose of the study treatment and had at least one PD measurement. A single IV dose was administered in SAD cohorts (designated Day 1), and multiple SC doses were administered in MAD cohorts (Day 1 and 29); the study duration including follow-up was 4–10 months from the screening. The plots presented are semi-logarithmic. *cTfh* circulating follicular T helper-like, *CXCR5* C-X-C chemokine receptor type 5, *IV* intravenous, *MAD* multiple ascending dose, *PD* pharmacodynamic*, SAD* single ascending dose, *SC* subcutaneous

B and cTfh cell counts generally showed dose-dependent reductions across cohorts (range of mean maximum depletion: 67.3–99.3% and 62.4–98.7%, respectively, in SAD cohorts, and 91.1–99.6% and 89.5–98.1%, respectively, in MAD cohorts; Fig. 3). The mean duration of B and cTfh depletion extended up to 71.6 and 62.0 days, respectively, in SAD, and 78.5 and 109.5 days, respectively, in MAD cohorts.

A total of 438 samples were analyzed for ADA, and 77 (17.6%) samples tested positive; these ADA-positive samples were further tested for NAb. Of these 77 samples, 60 (77.9%) samples tested NAb positive. In the IV SAD cohorts, a total of 23 ADA/NAb evaluable patients were analyzed, of these patients, seven (30.4%) tested positive for ADA and seven (30.4%) tested positive for NAb. In the SC MAD cohorts, a total of 31 ADA/NAb evaluable patients were analyzed; of these patients, 10 (32.3%) tested positive for ADA and seven (22.6%) tested positive for NAb.

No notable changes were seen in PK parameters, B and cTfh cell counts, and safety based on the ADA and NAb status of patients.

#### Exploratory (tertiary) pharmacodynamic and biomarker endpoints

To assess the mechanism of PF-06835375 and the functional effects of combination B and Tfh cell depletion, both primary neoantigen and recall vaccine responses were assessed. There were no changes in post- versus pre-vaccination B cell counts in participants who received placebo. In participants who received placebo, cTfh cell counts at Day 8 and Day 15 fluctuated around the baseline level (cTfh cell ratio range from 0.1 to 3) (Fig. S2 and S3). All study participants who received PF-06835375 had lower B cell counts compared to baseline after Day 1, with decreases to < 100 cells/µL in the lower doses and to < 10 cells/µL with higher doses, to Day 15. Almost all study participants who received PF-06835375 had lower cTfh cell ratios, which were ≤ 1 at Day 4 and remained low at Day 8 and Day 15. This observation confirms that the cTfh-inhibitory aspect of PF-06835375’s mechanism of action includes inhibition of cTfh cells in addition to its B cell-depleting properties, and indicates that PF-06835375 has the potential to reduce the number of newly formed autoreactive B cells and consequently autoantibody levels.

**Fig. 4:**
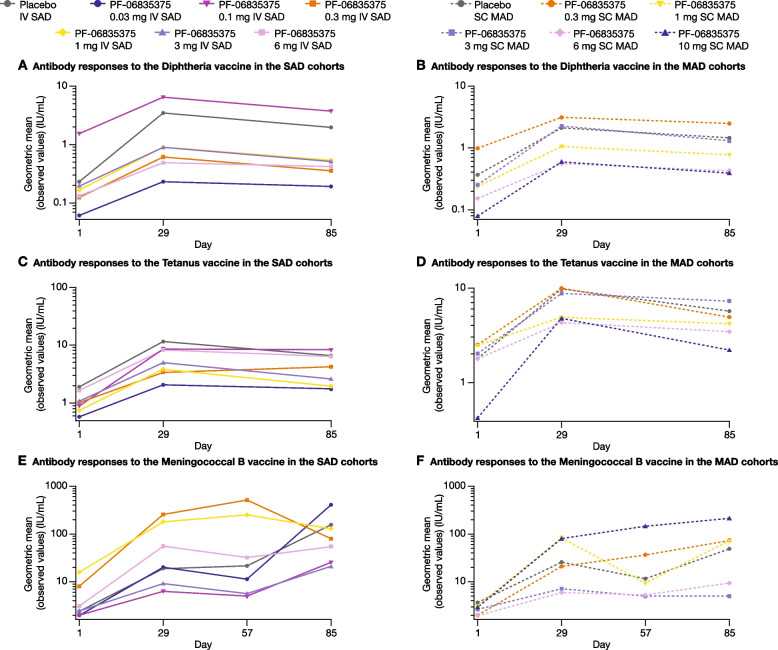
Geometric means of antibody responses to the **A**, **B**, **C**, **D** Tetanus/Diphtheria vaccine in the single ascending dose and multiple ascending dose cohorts and the **E**, **F** Meningococcal B vaccine in the single ascending dose and multiple ascending dose cohorts (PD analysis population). The PD analysis population included all patients who received at least one dose of the study treatment and had at least one PD measurement. Data were excluded from the plot if patients did not receive the Td or MenB vaccine at Day 4. If patients did not receive the MenB vaccine at Week 8, their Week 12 data were excluded from the plot. The lower limit of detection for anti-MenB was 4, a titer of 2 was reported in case of non-detectable MenB antibody. The plots presented are semi-logarithmic. *IV* intravenous, *MAD* multiple ascending dose, *MenB* Meningococcal B, *PD* pharmacodynamic*, SAD* single ascending dose, *SC* subcutaneous, *Td* tetanus/diphtheria

Regarding recall vaccine responses, the geometric means of diphtheria and tetanus antibody concentrations for the placebo group in both the SAD and MAD cohorts increased from Day 1 to Day 29, and were relatively stable from Day 29 to Day 85 (Fig. 4A–D). At Day 57, the geometric means of the MenB antibody concentrations showed increases in the recall responses in most treatment groups until Day 85 in both cohorts (Fig. 4E–F). Regarding neoantigen responses, the means of the MenB antibody concentrations in the placebo groups also increased from Day 1 to Day 29, and remained stable (SAD) or decreased (MAD) to Day 57 (Fig. 4E–F).

The geometric means of diphtheria and tetanus antibody concentrations for all the active treatment groups in both cohorts followed a similar trend to those of the placebo groups, increasing from Day 1 to Day 29 and remaining relatively stable from Day 29 to Day 85 (Fig. 4A–D). At Day 57, the geometric means of the MenB antibody concentrations showed increases overall until Day 85 for most active treatment groups in both cohorts (Fig. 4E–F). The geometric means of MenB antibody titers were seen to increase from Day 1 to Day 29 across all doses in both cohorts, and remained elevated until Day 57 (Fig. 4E and F). A trend in dose–response to the Td and the MenB vaccinations was not observed in patients in either of the SAD or MAD cohorts (Fig. 4A–F).

**Fig. 5:**
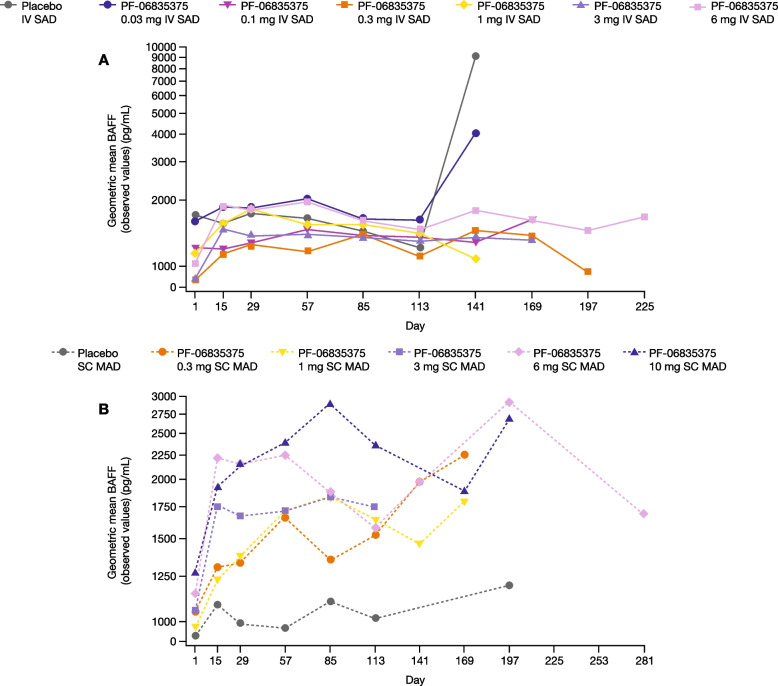
Geometric means of BAFF over time in the **A** single ascending dose and **B** multiple ascending dose cohorts (PD analysis population). The PD analysis population included all patients who received at least one dose of the study treatment and had at least one PD measurement. The plots presented are semi-logarithmic. *BAFF* B-cell activating factor, *IV* intravenous, *MAD* multiple ascending dose, *PD* pharmacodynamic*, SAD* single ascending dose, *SC* subcutaneous

Regarding serum BAFF, the geometric means at baseline were 1196.23 pg/mL and 1052.83 pg/mL in the SAD and MAD cohorts, respectively. The geometric means of BAFF over time are presented in Fig. 5A and B. Overall, BAFF levels increased over time, but there was some variation across doses. Increases were visible especially in the 0.03 mg SAD and placebo SAD cohort between Days 113 and 141, and in the MAD active treatment cohort across time points.

**Fig. 6:**
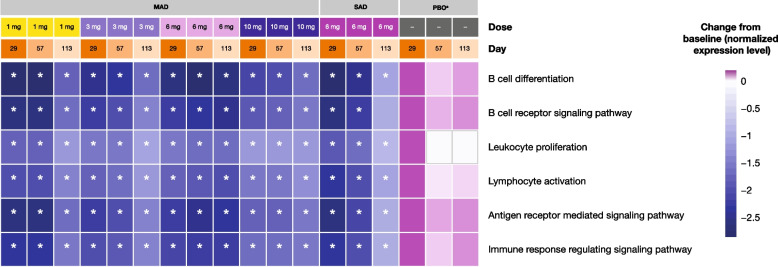
B cell-related pathways in patients in the multiple ascending and single ascending dose cohorts and the pooled placebo group. ^a^Patients receiving placebo in the MAD and SAD cohort were pooled into a single placebo group for analysis. Heatmap represents longitudinal modulation of gene sets upon treatment in 1, 3, 6, and 10 mg MAD and 6 mg SAD cohorts. The color scale depicts the range of change from baseline in normalized expression level for each gene set (a gene set is a group of genes represented by a gene ontology term shown to the right of the heatmap, in rows). “Day” and “Dose” legends provide column annotations for the heatmap. *FDR < 0.05. *FDR,* false discovery rate; *MAD* multiple ascending dose, *PBO* placebo, *SAD* single ascending dose

**Fig. 7:**
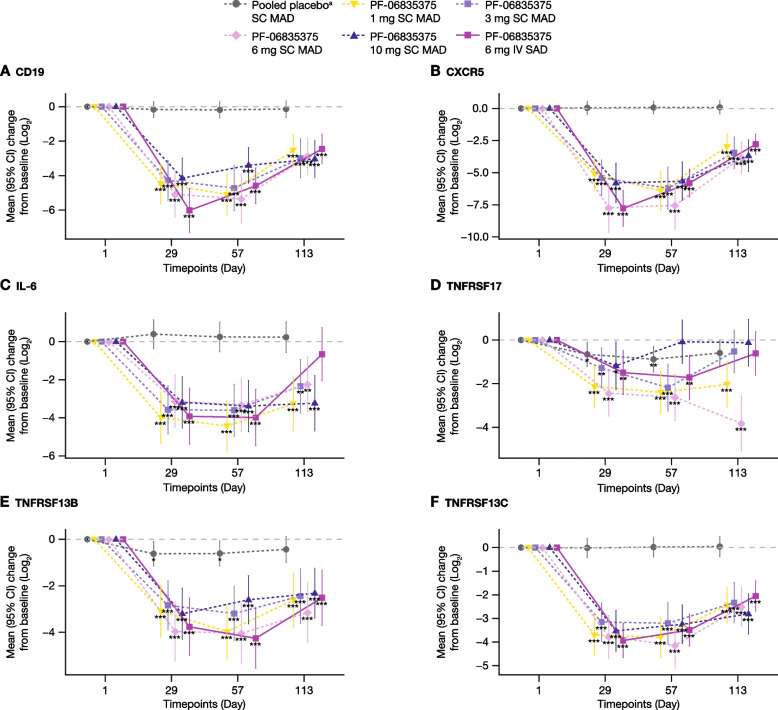
Mean (95% CI) change from baseline in the expression of B and Tfh cell-specific markers over time analyzed by RNA sequencing: **A** CD19, **B** CXCR5, **C** IL-6, **D** TNFRSF17, **E** TNFRSF13B, and **F** TNFRSF13C. ^a^Patients receiving placebo in the MAD and SAD cohort were pooled into a single placebo group for analysis. TNFRSF13B, TNFRSF13C, and TNFRSF17 encode transmembrane activator and calcium modulator and cyclophilin ligand interactor (TACI), B-cell activating factor receptor (BAFF-R), and B cell maturation antigen (BCMA) proteins, respectively. *CI,* confidence interval; *CXCR5* C-X-C chemokine receptor type 5, *IL* interleukin, *IV* intravenous, *MAD* multiple ascending dose, *SAD* single ascending dose, *TNFRSF* tumor necrosis factor receptor superfamily member. *p ≤ 0.05; **p ≤ 0.01; ***p < 0.001

Exploratory transcriptomic data generated using RNA-seq were analyzed for gene expression changes following B and cTfh cell depletion. The pathways that were most significantly affected at Days 29, 57, and 113 were B cell-related pathways/gene sets, which were downregulated in patients who received active treatment (Fig. 6; FDR < 0.05). Representative genes from pathways related to B-cell activation, differentiation, and receptor signaling showed a change in expression in samples from both SAD and MAD cohorts, whereas no significant changes were observed from baseline in the placebo group. Although changes in Tfh-specific gene signature remain to be determined, the expression of CXCR5 (B and Tfh cell surface marker) and CD19 (B cell surface marker) genes decreased significantly at all three time points assessed from Day 1 (Fig. 7). A significant decrease from baseline in the expression of the inflammatory cytokine gene IL-6 was seen across all doses and time points, with the exception of PF-06835375 6 mg IV in the SAD cohort at Day 113 (Fig. 7). Significant decreases in the expression of TNFRSF genes expressed on early-to-late B cell lineages TNFRSF17, TNFRSF13B, and TNFRSF13C were observed at most time points in patients treated with PF-06835375 (Fig. 7).

## Discussion

This first-in-human Phase 1 study evaluated the safety, tolerability, PK, and PD of PF‑06835375, an antibody against CXCR5, and its functional effect on vaccine responses in patients with seropositive SLE and RA. PF-06835375 was generally well tolerated in patients with seropositive SLE and RA; most TEAEs were mild or moderate in severity and only one patient discontinued due to a TEAE of disease progression in the placebo group. PD findings indicated potent and prolonged depletion of both CXCR5-positive populations of B and cTfh cells, but with intact vaccination responses across treatment groups and consistent with the mechanism of action of PF-0683575.

The TEAEs, including infections, and SAEs noted in this study were consistent with those observed with other B cell-depleting autoimmune therapies, such as constitutional symptoms [28] and infusion-induced side effects [29, 30]. In the current study, two patients receiving 3 mg IV had mild infusion-related reaction and one patient receiving 3 mg SC had mild allergic reaction, with the addition of steroids for the 3 mg and subsequent SC MAD cohorts. Notably, infections, overall, were the second most common TEAEs reported in the current study, including urinary tract infection, upper respiratory tract infection, and viral upper respiratory tract infection; all were mild or moderate in severity. The small number of serious infections reported in this Phase 1 study is comparable with that reported in a Phase 3 study of patients with RA receiving the T cell-targeted costimulatory modulator abatacept, among whom 1.9% developed serious infections (compared with 8.5% of patients receiving TNF inhibitor therapy with infliximab) [31], and a Phase 2b study in patients with RA receiving B-cell depleting therapy with rituximab, among whom 2.0% developed serious infections [32]. There were no reported events of herpes zoster or SARS-CoV-2 infection during the course of the study. The overall impact of the COVID-19 pandemic on this study was minimal, by extending the overall time span of the study. Mild or moderate abnormalities in ECGs were reported in three individuals, each resolving within the same day of dosing. Collectively, these findings did not identify safety concerns related to the B and Tfh cell depletion with PF-06835375.

In both the SAD and MAD cohorts of the current study, robust B- and cTfh-cell depletion were observed post-dose in all PF-06835375 groups, compared with patients receiving placebo. B cell counts in these patients markedly decreased starting at 8 h post-dose on Day 1. Additionally, B and Tfh cell depletion was similar in participants with RA or SLE. Sufficient and timely depletion of B cells may be important for the overall effectiveness of immunosuppressant agents given that, among patients with SLE or RA, complete B-cell depletion following rituximab infusion was associated with improved clinical responses over time compared with those whose B-cell depletion was incomplete at the same time point [33, 34]. We also observed that the cTfh cell absolute counts in all patients receiving higher doses of PF-06835375 (1, 3, and 6 mg IV in SAD cohorts; 3, 6, and 10 mg in MAD cohorts) markedly decreased at 8 h post-dose on Day 1; in MAD cohorts, low cTfh cell counts persisted then decreased again after the second study dosing on Day 29. Given that cTfh cells are reported to be elevated in both SLE and RA [20, 21], and promote the germinal center reaction and generation of autoantibody secreting plasma cells [19, 35], the reduction of cTfh cells in patients receiving higher doses of PF-06835375 has the potential to reduce disease activity.

All cohorts, except the lowest IV SAD doses of PF-06835375 (0.03 mg and 0.1 mg), had at least one ADA-positive patient; the median time of onset for treatment-induced ADA and NAb was 31.5 and 36.0 days, respectively. The immunogenicity data did not suggest any clinically relevant impact of PF-06835375 on PK, B and cTfh cell depletion, or safety.

The recall immune response of patients receiving PF-06835375 were assessed by Td vaccination, while MenB vaccination was used to assess neoantigen immune responses. These data demonstrate that patients receiving PF-0683575 exhibited intact humoral immune responses to vaccinations, despite potent B and cTfh cell depletion, and low post- versus pre-vaccination cTfh cell ratios, demonstrating patients receiving standard of care immunosuppressive agents were still able to mount a protective antibody response. Our data, regarding functional vaccine recall responses, are consistent with previous findings in belimumab-treated patients with SLE, among whom antibody responses to pneumococcal, tetanus, and influenza antigens were not reduced, indicating preservation of the memory B cell compartment [36]. Markedly, our data contrast with data from a Phase 2 study of patients with active RA and background methotrexate treated with rituximab, in which B-cell depletion reduced responses to neoantigen (keyhole limpet hemocyanin), but did not reduce recall responses to tetanus and diphtheria vaccines [37]. Our findings suggest that the ability of PF-06835375, at the exposures studied in this trial, to deplete B and cTfh cells did not appear to have a major impact on patients’ humoral immune responses to vaccines. However, it should be noted that the sample size was small, resulting in variability in the estimates of vaccine responses. Differences between our results and those reported previously may be attributed to differences in the study populations and background medications, and may be pertinent, given that treatment with immunosuppressants has been identified as a risk factor for inadequate responses to vaccines, including SARS-CoV-2 vaccination in patients with both SLE and RA [38, 39].

Regarding BAFF levels, observed increases over time have also been reported in studies using rituximab in patients with active RA and non-responders to anti-TNF-α, and in patients with active refractory SLE [40, 41].

RNA-seq data indicated that blood transcriptomic changes were dominated by significant decreases in the expression of B cell-related genes and pathways. While only a representative set of genes was depicted, B and Tfh cell depletion, and potential changes in inflammation-associated genes (as shown by IL-6), have been included in this manuscript. Decreased expression of the TNFRSF17 gene, which encodes B cell maturation antigen protein, primarily expressed on late-stage B cells, plasmablasts, and long-lived plasma cells [42], could be indicative of the effect of PF-06835375 in depleting late-stage, antibody-producing circulating B cells. Likewise, lower expression of TNFRSF13B and 13C genes, which respectively encode transmembrane activator and calcium modulator and cyclophilin ligand interactor and BAFF receptor proteins [43], could potentially dampen signaling-mediated B cell autoreactivity. A deeper investigation of the transcriptomic data could lead to a refined transcriptomic signature for PF-06835375 treatment, provide further insight into its mechanism of action, and allow for an assessment of disease-associated molecular changes. However, changes in protein levels remain to be addressed.

There have been many recent advances in therapies for autoimmunity, including B cell-targeted agents that build on approved approaches (i.e., CD20 depletion and BAFF inhibition). B-cell depletion with potent next-generation antibodies directed against CD20 along with both B- and CAR-T-cell therapies that specifically target CD19-expressing cells have emerged as novel approaches expanding into refractory patient populations [44, 45]. However, PF-06835375 is the first agent with dual function to target B cells and Tfh cells. Tfh cells play a critical role in immune responses by promoting B-cell development, germinal center formation, and antibody production. Aberrant proliferation and function of Tfh cells can lead to autoantibody production and development of autoimmune diseases [46, 47]. Although usually located in secondary lymphoid organs, Tfh cells can also be identified in human blood, and their frequency and phenotype are often altered in patients with autoimmune diseases including SLE and RA [46, 47]. Although the data in this manuscript show lower cTfh cell ratios post-dosing, there were still sufficient neoantigen and functional recall vaccination antibody responses, indicating that humoral immunity functionality remains preserved in participants receiving PF-06835375.

The strengths of this Phase 1 study include that it was conducted in patients with seropositive SLE and RA, and not in healthy volunteers, and that the majority of patients recruited to this study were female, which mirrors the global populations of patients with these conditions [2, 48, 49]. This study investigated a range of PF-06835375 doses and different administration routes, meaning that a variety of potential impacts on drug characteristics and PK were explored. A vaccination challenge was included for assessing the mechanism of action and safety, with respect to demonstrating the ability to produce neoantigen and functional recall responses to Td and MenB vaccinations over a range of B and Tfh cell depletion levels.

Limitations of this study include the small sample size and the lack of entry criteria requiring minimum disease activity, which may not be representative of the wider patient population seeking treatment. Also, the majority of patients were White, with a mean age of 53.3 years; prevalence rates for SLE are higher in non-White populations and RA has a bimodal age distribution with incidence peaks in the 25–45 and > 65 age groups [48, 50]. Furthermore, since the main aims of this Phase 1 study were to assess safety, tolerability, PK, and PD to evaluate the mechanistic aspects of PF-06835375. Efficacy was not evaluated as there was no minimum disease activity requirement for patients to enroll in the study. The lack of disease activity requirement for inclusion in the study limited the ability to interpret the effects of PF-06835375 on disease-related parameters. A Phase 2 study in primary immune thrombocytopenia (NCT05070845) is ongoing to explore clinical efficacy related to combination B- and Tfh-cell depletion.

## Conclusions

These first-in-human data support further development of PF-06835375 to assess the clinical potential for B and Tfh cell depletion as a treatment for autoimmune diseases.

### Supplementary Information

Additional file1: Fig. S1 Study design, Fig. S2 cTfh cell ratios by participant type in the single ascending dose cohort (PD analysis population), Fig. S3 cTfh cell ratios by participant type in the multiple ascending dose cohort (PD analysis population). Overview of study design for Part 1 and Part 2, cTfh cell ratios by participant type in the single and multiple ascending dose cohorts (PD analysis population).

## Data Availability

Upon request, and subject to review, Pfizer will provide the data that support the findings of this study. Subject to certain criteria, conditions, and exceptions, Pfizer may also provide access to the related individual de-identified participant data. See https://www.pfizer.com/science/clinical-trials/trial-data-and-results for more information.

## Abbreviations

ACR: American college of rheumatology
ADA: Anti-drug antibodies
AE: Adverse event
ANA: Anti-nuclear antibodies
Anti-dsDNA: Anti-double-stranded deoxyribonucleic acid
AUCinf: Area under the concentration–time profile from time 0 extrapolated to infinite time
AUClast: Area under the concentration–time profile from time 0 to the time of the last quantifiable concentration
AUC_tau_: Area under the serum concentration–time profile from time 0 to time tau, the dosing interval, where tau = 28 days
BAFF: B-cell activating factor
BAFF-R: B-cell activating factor receptor
BMI: Body mass index
CL: Clearance
CL/F: Apparent clearance
C_max_: Maximum serum concentration during the dosing interval
CPM: Counts per million
cTfh: Circulating follicular T helper cell
CXCR5: C-X-C chemokine receptor type 5
hsCRP: High-sensitive C-reactive protein
ECG: Electrocardiogram
EULAR: European alliance of associations for rheumatology
FDR: False discovery rate
FGSEA: Fast gene set enrichment analysis
Ig: Immunoglobulin
IL: Interleukin
IFN-γ: Interferon-gamma
IV: Intravenous
MAD: Multiple ascending dose
MenB: Meningococcal B
NAb: Neutralizing antibodies
NOAEL: No observed adverse effect level
PCA: Principal component analysis
PD: Pharmacodynamics
PK: Pharmacokinetics
RA: Rheumatoid arthritis
RNA-seq: RNA sequencing
SAD: Single ascending dose
SAE: Serious adverse event
SC: Subcutaneous
SLE: Systemic lupus erythematous
SLEDAI-2K: Systemic Lupus Erythematosus Disease Activity Index 2000
SLICC: Systemic Lupus International Collaborating Clinics
t_1/2_: Terminal half-life
Td: Tetanus/Diphtheria
TEAE: Treatment-emergent adverse event
Tfh: Follicular T helper
T_max_: Time for C_max_
TNF: Tumor necrosis factor
TNFRSF: Tumor necrosis factor receptor superfamily
