# Supplementary material for: A Phase 1, randomized, double-blind, placebo-controlled, single- and multiple-dose escalation study to evaluate the safety and pharmacokinetics/pharmacodynamics of PF-06835375, a C-X-C chemokine receptor type 5 directed antibody, in patients with systemic lupus erythematosus or rheumatoid arthritis
Source: Arthritis Res Ther. 2024 Jun 6;26:117. doi: 10.1186/s13075-024-03337-2 (PMC11155132; doi:10.1186/s13075-024-03337-2)
Supplement: Supplementary file 1 — Additional file1: Fig. S1 Study design, Fig. S2 cTfh cell ratios by participant type in the single ascending dose cohort (PD analysis population), Fig. S3 cTfh cell ratios by participant type in the multiple ascending dose cohort (PD analysis population). Overview of study design for Part 1 and Part 2, cTfh cell ratios by participant type in the single and multiple ascending dose cohorts (PD analysis population). [file 13075_2024_3337_MOESM1_ESM.docx]

**Fig. S1** Study design

**
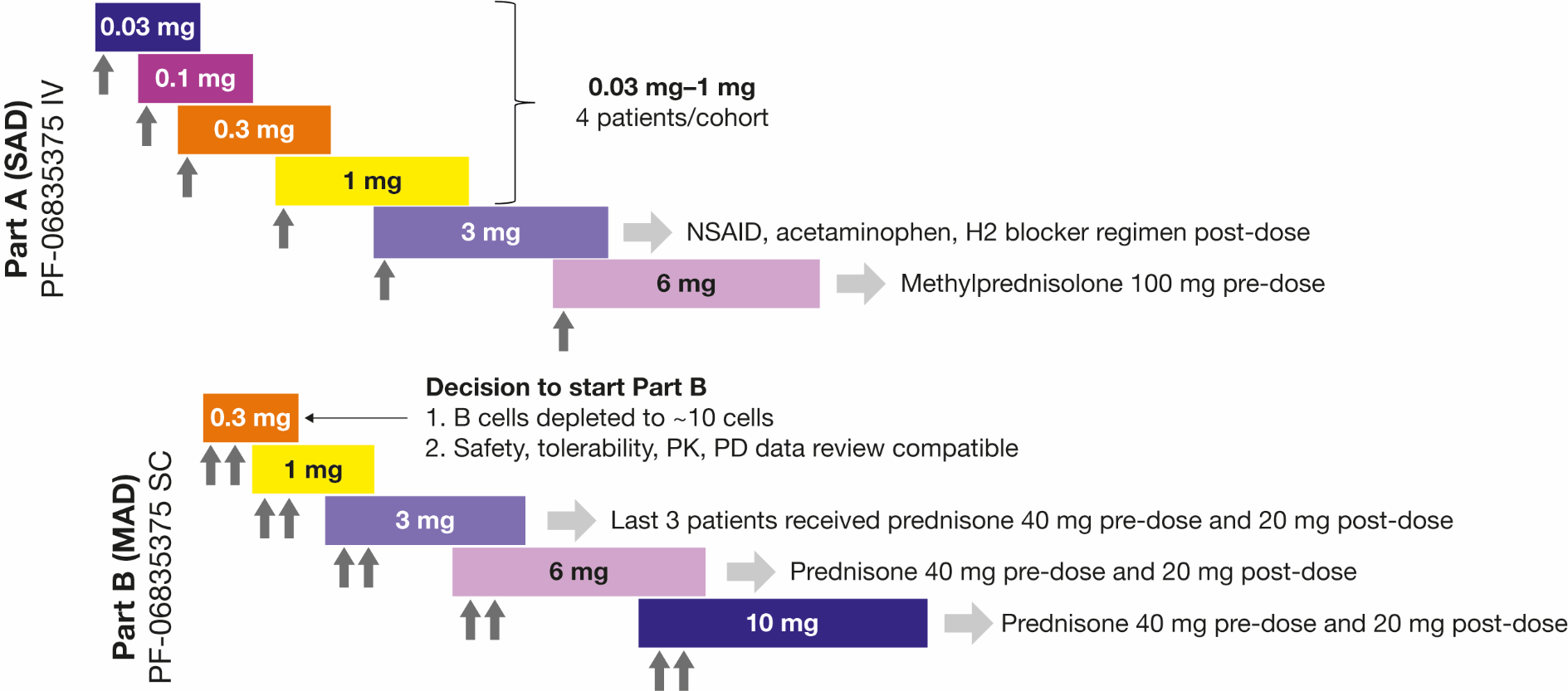
**

Cohorts in Part A were initiated sequentially with participants receiving SAD doses of 0.03, 0.1, 0.3, 1, 3, or 6 mg IV PF-06835375. Patients in Part B received two doses of 0.3, 1, 3, 6, or 10 mg SC PF‑06835375 (first dose on Day 1, second dose on Day 29); patients in Part B were enrolled concurrently with Part A following completion of the initial cohort in Part A. Arrows indicate SAD and MAD dosing schedules
*MAD* multiple ascending dose, *NSAID*, nonsteroidal antiinflammatory drug; *SAD* single ascending dose

**Fig. S2** cTfh cell ratios by participant type in the single ascending dose cohort (PD analysis population)


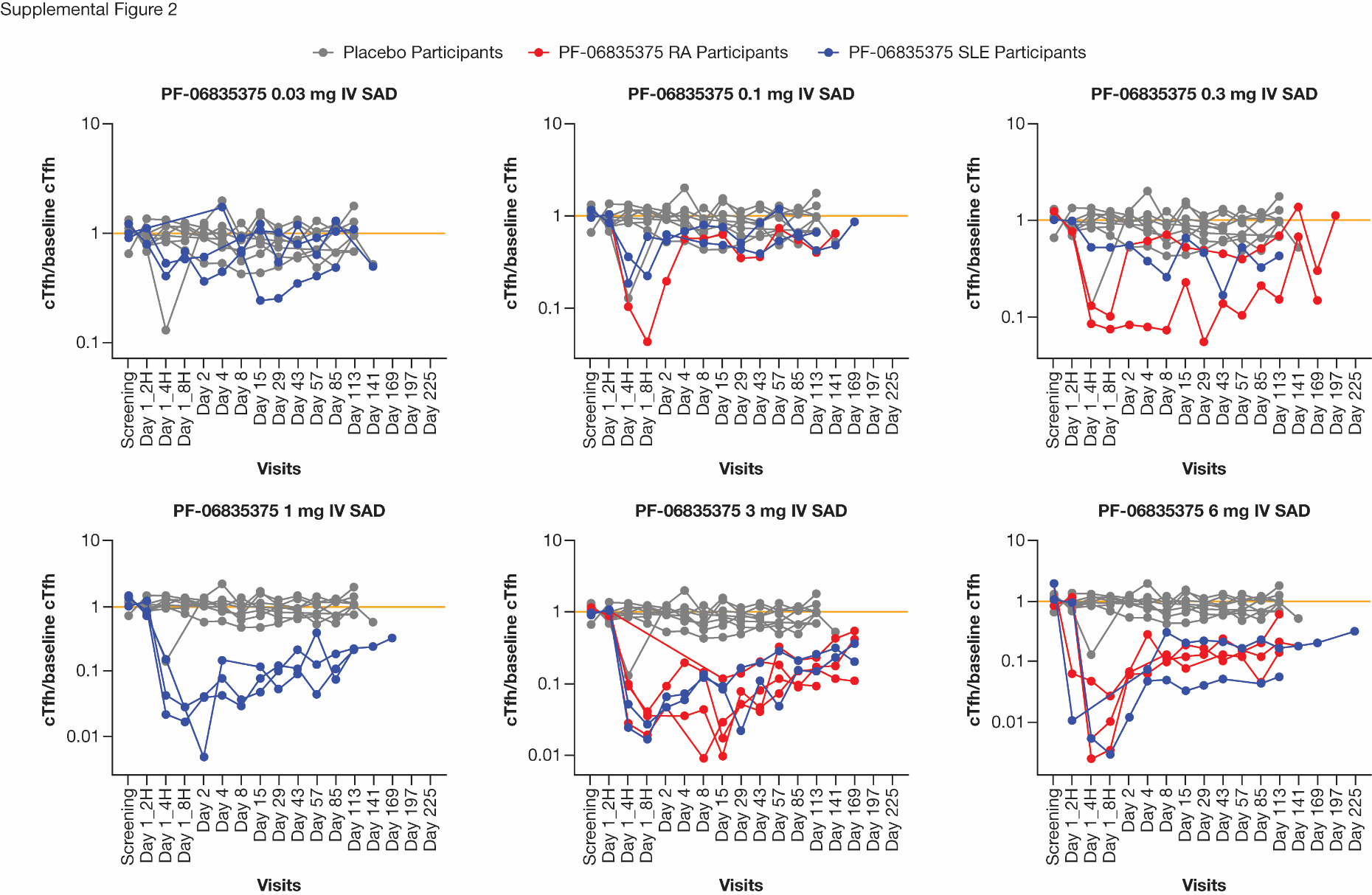


*cTfh* circulating follicular T helper-like, *IV* intravenous, *PD* pharmacodynamic*, RA* rheumatoid arthritis, *SAD* single ascending dose, *SLE* systemic lupus erythematosus

**Fig. S3** cTfh cell ratios by participant type in the multiple ascending dose cohort (PD analysis population)


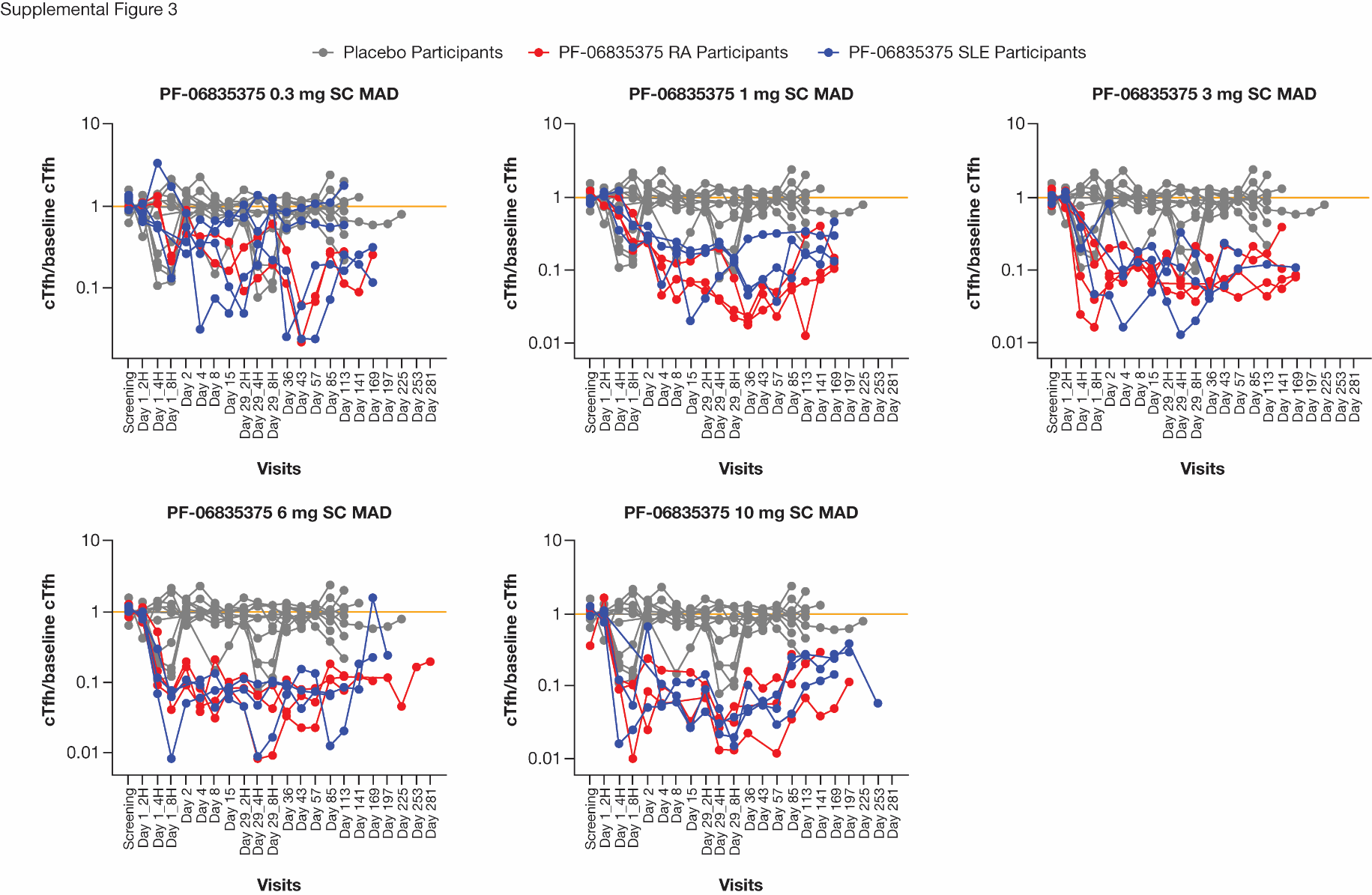


*cTfh* circulating follicular T helper-like, *MAD* multiple ascending dose, *PD* pharmacodynamic*, RA* rheumatoid arthritis, *SLE* systemic lupus erythematosus,

*SC* subcutaneous
